# The Bioflavonoids Rutin and Rutin Succinate Neutralize the Toxins of *B. jararaca* Venom and Inhibit its Lethality

**DOI:** 10.3389/fphar.2022.828269

**Published:** 2022-02-21

**Authors:** Ana Teresa Azevedo Sachetto, Jackson Gabriel Miyamoto, Alexandre Keiji Tashima, Ana Olívia de Souza, Marcelo Larami Santoro

**Affiliations:** ^1^ Laboratory of Pathophysiology, Institute Butantan, São Paulo, Brazil; ^2^ Department of Medical Sciences, School of Medicine, University of São Paulo, São Paulo, Brazil; ^3^ Department of Biochemistry, Escola Paulista de Medicina, Federal University of São Paulo, (EPM/UNIFESP), São Paulo, Brazil; ^4^ Laboratory of Development and Innovation, Institute Butantan, São Paulo, Brazil

**Keywords:** coagulation, hemostasis, rutin, snake venom, antivenom

## Abstract

The venom of the Brazilian pit viper *Bothrops jararaca* (BjV) is a complex mixture of molecules, and snake venom metalloproteinases (SVMP) and serine proteinases (SVSP) are the most abundant protein families found therein. Toxins present in BjV trigger most of the deleterious disturbances in hemostasis observed in snakebites, i.e., thrombocytopenia, hypofibrinogenemia and bleedings. The treatment of patients bitten by snakes still poses challenges and the bioflavonoid rutin has already been shown to improve hemostasis in an experimental model of snakebite envenomation. However, rutin is poorly soluble in water; in this study, it was succinylated to generate its water-soluble form, rutin succinate (RS), which was analyzed comparatively regarding the chemical structure and characteristic features of rutin. Biological activities of rutin and RS were compared on hemostatic parameters, and against toxic activities of crude BjV *in vitro*. *In vivo*, C57BL/6 mice were injected i.p. with either BjV alone or pre-incubated with rutin, RS or 1,10-phenanthroline (o-phe, an SVMP inhibitor), and the survival rates and hemostatic parameters were analyzed 48 h after envenomation. RS showed the characteristic activities described for rutin – i.e., antioxidant and inhibitor of protein disulfide isomerase – but also prolonged the clotting time of fibrinogen and plasma *in vitro*. Differently from rutin, RS inhibited typical proteolytic activities of SVMP, as well as the coagulant activity of BjV. Importantly, both rutin and RS completely abrogated the lethal activity of BjV, in the same degree as o-phe. BjV induced hemorrhages, falls in RBC counts, thrombocytopenia and hypofibrinogenemia in mice. Rutin and RS also improved the recovery of platelet counts and fibrinogen levels, and the development of hemorrhages was totally blocked in mice injected with BjV incubated with RS. In conclusion, RS has anticoagulant properties and is a novel SVMP inhibitor. Rutin and RS showed different mechanisms of action on hemostasis. Only RS inhibited directly BjV biological activities, even though both flavonoids neutralized *B. jararaca* toxicity *in vivo*. Our results showed clearly that rutin and RS show a great potential to be used as therapeutic compounds for snakebite envenomation.

## Introduction

Snakebite envenomation is a priority among the tropical neglected diseases recognized by the World Health Organization (WHO) (World Health Organization, 2021). In Brazil, *Bothrops* snakes stand out for being responsible for 90% of the snakebite accidents. Acknowledging that, *Bothrops jararaca* (*B. jararaca*) is considered by WHO as a species with high medical relevance (World Health Organization, 2021; Sistema de Informação de, 2022).

Snake venom is used for predation and defense, which led to a natural evolution toward a high complexity and specificity (Gutiérrez et al., 2017). *Bothrops* venom is mainly composed by proteins, as snake venom metalloproteinases (SVMP), serine proteases (SVSP), phospholipases A_2_ (PLA_2_), C-type lectins, L-amino acid oxidases, hyaluronidases, among others (Koh et al., 2006; Deolindo et al., 2010; Sajevic et al., 2011; Takeda et al., 2012; Izidoro et al., 2014). These proteins are responsible for the toxic and lethal activities of the venom, which induce an inflammatory response, oxidative/nitrosative stress and hemostatic disturbances, as thrombocytopenia, consumptive coagulopathy and bleedings (Kamiguti et al., 1986; Maruyama et al., 1990; Santoro et al., 1994; Barraviera et al., 1995; Gonçalves and Mariano, 2000; Gutiérrez and Rucavado, 2000; Petricevich et al., 2000; Avila-Agüero et al., 2001; Sano-Martins et al., 2003; Schattner et al., 2005; Santoro et al., 2008a; Santoro et al., 2008b; Zychar et al., 2010; Moura-da-Silva and Baldo, 2012; Ferraz et al., 2015; Gutiérrez et al., 2017).

Although snakebite envenomation is a complex disease, its only official and prescribed treatment is the administration of animal-derived antivenoms, which directly inhibits venom toxins. *Bothrops* antivenom is used to treat patients bitten by *Bothrops* snakes, but it has limitations to block the snakebite-induced local effects and secondary complications as oxidative/nitrosative stress (Cardoso et al., 1993; Jorge et al., 1995; Battellino et al., 2003; Strapazzon et al., 2014). Therefore, studies have been conducted aiming to search for and develop new complementary medicinal products.

Several polyphenols from plant sources were shown to have beneficial activities for human and animal health (Selamoglu, 2017). Flavonoids are included on the class of polyphenols and were already tested to combat snake venom effects on victims (Castro et al., 1999; Nishijima et al., 2009; Pithayanukul et al., 2010). Among the several compounds with therapeutic potential, quercetin-3-rutinose (rutin) was elected for being a compound derived from plants, easily accessible, with low-cost and a broad range of activities, and can be produced in several pharmaceutical formulations (Behling et al., 2004; Pietta, 2000; Kandaswami and Middleton, 1994; Afanas’ev et al., 1989; Ganeshpurkar and Saluja, 2017). Rutin is a flavonoid which presents the core structure of a quercetin (3,5,7,3′-4′-pentahydroxyl flavone) and two glycosides at the 3′ position of the C ring. However, the use of rutin shows limitations regarding its aqueous solubility and bioavailability. As alternatives for these challenges, either using compounds analogous to rutin (as isoquercetin and troxerutin), or chemically modifying rutin to improve its solubility – e.g., by conjugation of rutin with metal ions or succinylation of hydroxyl groups in rutin glycosides (Krewson and Couch, 1952; Alluis et al., 2000; Pedriali et al., 2008a; Gullón et al., 2017) – have been tested.

Rutin exhibits different activities, as an antioxidant, anti-inflammatory, antithrombotic, pro-hemostatic and protein disulfide isomerase (PDI) inhibitor (Afanas’ev et al., 1989; Ganeshpurkar and Saluja, 2017; Choi et al., 2015; Jasuja et al., 2012; Stopa et al., 2017; Schulman et al., 2016; Sharma et al., 2013; Panche et al., 2016; Kauss et al., 2008; Kirchner et al., 2013). This turns rutin into a promising therapeutic agent against complex diseases as snakebite envenomation. In fact, rutin and analogous compounds have been studied for their properties against snake venoms for more than 7 decades (Seba, 1949). However, a recent study from our group (Sachetto et al., 2018) showed that rutin potential towards snake envenomation is much broader than previously observed. Therefore, we aimed to synthetize and characterize a water-soluble form of rutin, named rutin succinate (RS), and investigate the possible inhibitory effects of both rutin and RS on venom toxins activities *in vitro* and *in vivo*.

## Material and Methods

### Materials

Lyophilized venom from adult specimens of *B. jararaca* snakes was obtained from the Laboratory of Herpetology, Instituto Butantan (Sistema Nacional de Gestão do Patrimônio Genético e do Conhecimento Tradicional Associado, SisGen AF375C2). *Bothrops* antivenom was kindly donated by Instituto Butantan (lot: 1305077). Di-eosin-GSSG and purified PDI were kindly donated by Dr. Francisco Laurindo, INCOR, USP. Rutin (code R5143), succinic acid, succinic anhydride, 1,10-phenantroline (o-phe), AEBSF, bovine fibrinogen, porcine skin gelatin, casein, collagenase from *Clostridium histolyticum*, and bacitracin A were obtained from Sigma-Aldrich (United States). All other reagents were of analytical grade or better.

### Animals

Male C57BL/6 mice, weighing 18–22 g, were obtained from the Animal Facility of Instituto Butantan, and were maintained with free access to food and water. Experimental procedures involving mice were in accordance with National Guidelines, the Conselho Nacional de Controle de Experimentação Animal (CONCEA) and were approved by the Institutional Animal Care and Use Committees (Instituto Butantan, 4491070319 and Faculdade de Medicina da Universidade de São Paulo, 1334/2019).

### Rutin Succinate Synthesis

**FIGURE 1:**
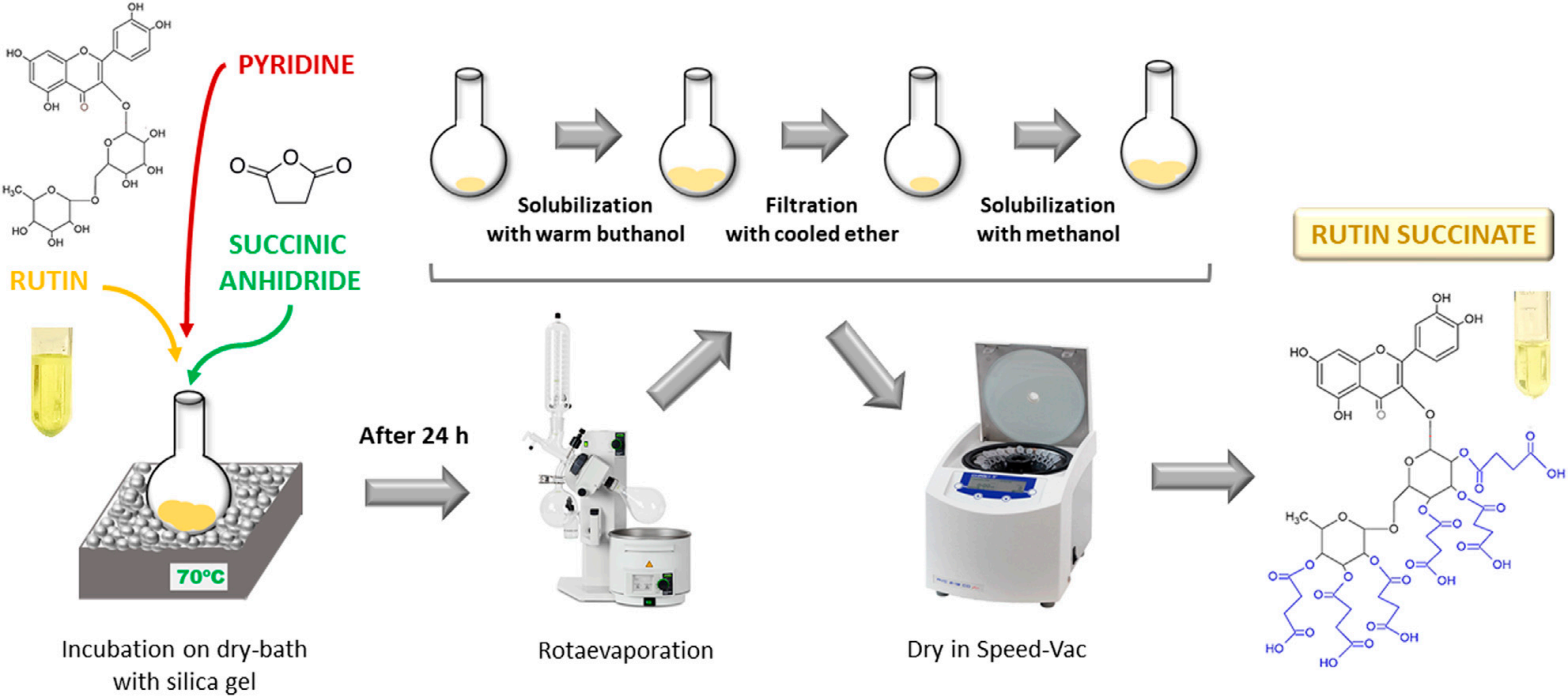
Schematic representation of rutin succinate synthesis.

Rutin succinate (RS) was synthetized by succinylation of the hydroxyl groups present in the sugar moieties of rutin (Pedriali et al., 2008b) (Figure 1). A solution of rutin (2.5 g), succinic anhydride (3.75 g) and pyridine (100 ml) was heated at 70°C por 24 h in a dry-bath. The reaction product (RS) was rotaevaporated (rotaevaporator R-210, Buchi) to remove pyridine, solubilized in warm butanol, cooled and filtered with cooled diethyl ether. Organic solvents were removed by rotaevaporation and the final product (RS) was solubilized in methanol and totally dried in a Speed-Vac equipment (RVC 2-18 CD Plus, Christ) and stored at room temperature.

### Characterization of RS

#### UV-Visible Spectrophotometry

UV-visible spectrophotometry was utilized to verify if the core flavonoid chemical structure of RS was intact following rutin succinylation. Solutions of rutin and RS (205 µM in methanol) were analyzed in a quartz 96-well microplate (Molecular Devices, United States) at a spectrum of 240 nm–450 nm in a microplate reader (SpectraMax 190, Molecular Devices).

#### High Performance Liquid Chromatography

The chromatographic analysis of RS and rutin was performed by reversed-phase high performance liquid chromatography (HPLC) [Äkta Purifier 10 HPLC system, GE (Cytiva)]. For that, aliquots (20 µl) of rutin or RS (5 mg/ml in methanol) were loaded into a C18 column (ACE, 250 × 4.6 mm), and eluted at a flow rate of 1 ml/min, using a gradient from 45 to 70% B (mobile phases A: 99.9% H_2_O/0.1% trifluoroacetic acid (TFA) and B: 90.0% methanol/9.9% H_2_O/0.1% TFA) during 25 min. The absorbance UV/Vis detector was set to the wavelengths of 214, 254, and 280 nm. The analytical profiles of succinic anhydride (10 µl of a 5 mg/ml solution), succinic acid (10 µl of a 5 mg/ml solution), pyridine (3 µl) and diethyl ether (40 µl) were obtained and compared to that of the RS preparations.

#### Mass Spectrophotometry (LC-MS/MS)

Liquid chromatography-mass spectrometry (LC-MSE) analyses were performed on a Synapt G2 HDMS mass spectrometer (Waters) coupled to the chromatographic system nanoAcquity UPLC (Waters). Rutin and RS were dissolved in methanol and diluted in formic acid 0.1% to a final concentration of 10 ng/µl and 100 ng/µl, respectively. Samples of rutin and RS (10 and 500 ng respectively) were loaded into a trap column (Acquity UPLC M-Class Symmetry C18 Trap Column, 100 Å, 5 μm, 300 μm × 25 mm, Waters) at 8 µl/min in phase A (0.1% formic acid) for 5 min. Samples were subsequently eluted in an analytical column (Acquity UPLC M-Class HSS T3 Column, 1.8 μm, 300 μm × 150 mm, Waters) with a gradient of 20%–60% of phase B (0.1% formic acid in ACN) over 15 min at a flow rate of 3 µl/min. Analyses were performed in the data independent acquisition (DIA) method MSE (Silva et al., 2006; Abreu et al., 2017), in the m/z range of 50–2,000 and set up in resolution mode. Precursor ions were fragmented by collision-induced dissociation (CID), switching from low (4 eV) to high (ramped from 20 to 30 eV) collision energy, for accurate measurement of both precursor and fragment ions. Scan times were set to 1.0 s. The ESI source was operated in the positive mode with a capillary voltage of 3.0 kV, block temperature of 100°C, and cone voltage of 15 V. The column temperature was set at 55°C. For lock mass correction, a [Glu1]-Fibrinopeptide B solution (500 fmol/ml in 50% methanol, 0.1% formic acid; Peptide 2.0) was infused through the reference sprayer at 2 μl/min and sampled every 60 s for external calibration (Pedroso et al., 2017).

#### Total Antioxidant Capacity

The total antioxidant capacity (TAC) of rutin and RS was assayed based on the CUPRAC colorimetric method (Ribeiro et al., 2011; Sachetto et al., 2018). The final concentrations of rutin and RS in the assay were 0.015, 0.029, 0.059, and 0.118 mM. As a standard curve, 2-fold serially dilutions of reduced L-glutathione (GSH), ranging from 12.5 to 300 μM, were used. Results were expressed as mM of GSH.

#### Quenching of Calcium, Magnesium and Zinc Ions

The ability of rutin and RS to quench divalent metal ions was tested using commercial assays (Magnesium kit for MgCl_2_ and ZnCl_2_, and Ca arsenazo Liquiform kit for CaCl_2_, Labtest, Brazil). Rutin, RS and succinic acid (SA, free form of succinic anhydride) were two-fold serially diluted (using 1% DMSO in saline) and added to the standards (2 mg/ml MgCl_2_, 25 mM ZnCl_2_ or 10 mg/ml CaCl_2_). The assays were carried out following manufacturer’s instructions and the final concentration of rutin, RS or succinic acid were: 1.11, 2.23, 4.46, 8.92, and 17.84 µM (calcium assay); 0.26, 0.52, 1.04, 2.08, and 4.15 µM (magnesium assay); and 31.25, 62.5, 125, 250, and 500 µM (zinc assay).

#### Analysis of PDI Structure and Activity

PDI structure was analyzed testing the intrinsic fluorescence of tryptophan residues (Trp) (Ado et al., 2006). In 96-well black microplates (Costar, United States), a PDI solution (300 nM, 50 µl/well) and 50 µl of rutin or RS solutions (0.59 mM in Tris-HCl buffer (20 mM Tris, 140 mM NaCl, 1 mM CaCl_2_, pH 8.0 and 1% DMSO). After 15 min of incubation at room temperature, the fluorescence spectrum was recorded (excitation. 280 nm, and emission: 360–510 nm). PDI reductase activity were determined using di-eosin-GSSG probe as previously described (Raturi and Mutus, 2007; Montano et al., 2014; Oliveira et al., 2019). Rutin or RS was diluted in 1% DMSO in phosphate buffer to final concentrations of 60 µM. Results were expressed as relative fluorescence units (RFU) and Vmax were also analyzed.

### Activities of Rutin and RS on Hemostatic Parameters *In Vitro*

#### Prothrombin Time and Activated Partial Thromboplastin Time

Commercial kits for prothrombin time (DiaPlastin, DiaMed, Brazil) and aPTT (TTPA CLOT, Bios Diagnostica, Brazil) were used according to manufacturer’s instructions. Mouse plasma was diluted in saline (1:10 for PT and 1:5 for aPTT) and pre-incubated with rutin or RS solutions for 10 min at 37°C before the assays. Rutin and RS were two-fold serially diluted in 154 mM NaCl solution containing 1% DMSO.

#### Thrombin Time

Thrombin time assay was performed by pre-incubating 100 µl of mouse plasma [diluted 1:4 in Tyrode buffer (137 mM NaCl, 2.7 mM KCl, 3 mM NaH_2_PO_4_, 10 mM HEPES, 5.6 mM dextrose, 1 mM MgCl_2_, 2 mM CaCl_2_, pH 7.4)] or bovine fibrinogen solution (4 mg/ml in Tyrode buffer) with 12.5 µl of Tyrode buffer or solutions of rutin, RS or SA for 15 min at 37°C. After incubation, 12.5 µl of thrombin-calcium solution (10 U/ml thrombin and 100 mM CaCl_2_) were added, and the clotting time was determined until 300 s in a coagulometer (STart 4, Diagnostica Stago). The final concentrations of rutin, RS and SA were 0.18, 0.37, 0.74, 1.47, and 2.95 mM, respectively.

#### Absorbance and Fluorescence Profile of Fibrinogen and Albumin

Rutin, RS and SA were two-fold serially diluted as described previously for thrombin time. The formation of fibrinogen complexes was tested analyzing the turbidity of fibrinogen solutions (Zhang et al., 2013). In a quartz 96-well microplate, aliquots (50 µl/well) of a fibrinogen solution (6.4 mg/ml in PBS, pH 7.4), followed by addition of aliquots (50 µl/well) of PBS, rutin, RS or SA. Absorbances were read at 405 nm. Moreover, the structure of fibrinogen and bovine serum albumin (BSA) were analyzed by the addition of fibrinogen or BSA solutions diluted in Tyrode buffer (0.8 mg/ml, final concentration), and pre-incubation with rutin, RS or SA solutions for 15 min at 37°C. The fluorescence of Trp was measured as described above.

### Activities of Rutin and RS on BjV Activities *In Vitro*

The following experiments were performed after pre-incubations of BjV with buffer containing DMSO (control of reaction), rutin, RS or SA for 30 min at 37°C. Different concentrations of rutin, RS and SA were used, depending on the concentration of BjV, with a range of 2.25, 4.5, 9, 18, and 36 times the amount of BjV. As positive controls of inhibition, BjV was pre-incubated with 8 mM AEBSF (inhibitor of SVSP), and/or 13 mM o-phe or Na_2_EDTA (inhibitors of SVMP) for 1 h at 37°C (Yamashita et al., 2014). The results were calculated as percentage of activity based on BjV (considered 100% of activity) if not specified otherwise.

#### Analysis of BjV and Jararhagin Structure

The fluorescence of Trp in BjV proteins and jararhagin (an important SVMP isolated from BjV) (Moura-da-Silva and Baldo, 2012) was analyzed as described above. BjV or jararhagin was diluted in Tris-HCl buffer (20 mM Tris, 140 mM NaCl, 1 mM CaCl_2_, pH 8.0), both in final concentration of at 0.2 mg/ml, and incubated with solutions of rutin, RS or SA two-fold serially diluted in Tris-HCl buffer (containing 1% DMSO), with final concentrations ranging from 0.07375 to 1.18 mM.

#### L-Amino Acid Oxidase, Hyaluronidase and SVSP Activities

The activities of LAAO, hyaluronidases and SVSP were analyzed using L-leucine, hyaluronic acid and BAPNA as substrates, respectively (Antunes et al., 2010; Sachetto et al., 2018). BjV was diluted on the specific assay buffers, so that the final concentrations of BjV in the assays were 17 µg/ml for LAAO, 66 µg/ml for hyaluronidases, and 44 µg/ml for SVSP. Rutin, RS and SA were diluted in the same buffer as BjV with the addition of DMSO and final concentrations ranging from 0.23 to 2.69 mM (LAAO), 0.18 mM–3.9 mM (hyaluronidases) and 0.13–2.01 (SVSP) were tested.

#### Activities of SVMP

SVMP were tested regarding their collagenolytic, gelatinolytic, caseinolytic, fibrinogenolytic and activator of prothrombin activities (Antunes et al., 2010; Sachetto et al., 2018). For the collagenolytic activity, azocoll collagen (Antunes et al., 2010; Sachetto et al., 2018) was incubated with 0.072 mg/ml BjV and rutin, RS or SA, at final concentrations ranging from 0.11 to 1.76 mM. The gelatinolytic, caseinolytic and fibrinogenolytic activity (Santoro and Sano-Martins, 1993; Antunes et al., 2010) was determined by the analysis of the degradation profile of intact proteins by SDS-PAGE, using mixtures of BjV (17 µg/ml, final concentration), and rutin, RS or SA at final concentrations ranging from 0.07 to 1.13 mM. The degradation of fibrinogen and gelatin was also tested using jararhagin and collagenase from *C. histolyticum* (38 µg/ml, Sigma), respectively. The densitometric analysis was performed using the software ImageJ (Fiji), and differences between the intact proteins and the proteins incubated with BjV was considered as 100% of activity. The prothrombin activating activity was determined as described previously (Yamada and Morita, 1997; Antunes et al., 2010), using BjV at 0.8 µg/ml (final concentration), and rutin, RS or SA at final concentrations ranging from 3.03 to 48.49 mM.

#### Coagulant Activity

The coagulant activity of BjV was tested using mouse plasma or bovine fibrinogen using a modification of the minimum coagulant dose technique. BjV was used at 50 µg/ml, and rutin, RS or SA at 0.18 mM–2.95 mM (final concentrations).

### *In Vivo* Experimental Groups and Procedures

BjV, RS or SA were dissolved in sterile saline and rutin was dissolved in equal parts of propylene glycol and sterile saline. Two doses of BjV were utilized: 3.2 mg/kg b.w. i.p., i.e., 2× lethal dose 50% of BjV (LD_50_, moderate envenomation), and 4.8 mg/kg b.w. i.p. (3×LD_50_, severe envenomation) (Antunes et al., 2010). Rutin and RS were utilized at doses 9 times higher than BjV (28.8 or 43.2 mg/kg b.w. i.p.), as previously described (Sachetto et al., 2018). To better understand RS activities, SA was used at 9.6 mg/kg b.w. i.p. (same molarity as rutin and RS). SVMP or SVSP were inhibited with the use of 13 mM o-phe or 8 mM AEBSF, respectively. Bacitracin A (80 mg/kg p.v. i.p.) was used to verify the contribution of thiol isomerases in the severe envenomation (Cho et al., 2008). BjV was pre-incubated with these solutions and injected into animals. Male C57BL/6 mice (18 g–22 g) were randomly allocated in different experimental groups: saline (negative control), BjV (positive control), BjV + rutin, BjV + RS, BjV + SA, BjV + AEBSF, BjV + o-phe and BjV + bacitracin A.

#### Survival Analysis

The survival of mice was analyzed after injection of 3× LD_50_ of BjV alone [severe envenomation, (Antunes et al., 2010)] or pre-incubated with rutin RS, SA, o-phe, or bacitracin A. The number of surviving animals was recorded at each hour from 1 to 9 h, and at 24 and 48 h after the injection of solutions.

#### Blood and Organ Collection

After 48 h, blood was collected from surviving mice and used for complete blood cell counts (CBC) and plasma collection, as described previously (Sachetto et al., 2018). For histological analysis, fragments of liver, kidney, lungs, heart, pancreas, spleen, intestine, diaphragm and intestinal wall from dead or euthanized mice were collected, submersed on Bouin’s solution until processing of histological material.

#### Hemostatic Parameters

CBC was performed in an automated cell counter BC-2800 Vet (Mindray, China). Plasma fibrinogen was measured in citrated plasma using a colorimetric assay (Ratnoff and Menzie, 1951; Sachetto, 2018).

#### Statistical Analysis

Normal distribution and homoscedasticity of the results were analyzed using the software STATA™, version 10, and data were transformed whenever necessary. One-way or Kruskal-Wallis test was used, followed by *post-hoc* tests (Bonferroni or Dunn’s tests). The software SPSS (version22), Sigma Plot (version 12.0) and R (version 4.00) were employed for these analyses. Data was considered statistically significant when *p* < 0.05, and results were expressed as mean ± standard error of mean (SEM).

## Results

### RS Synthesis and Characterization

**FIGURE 2:**
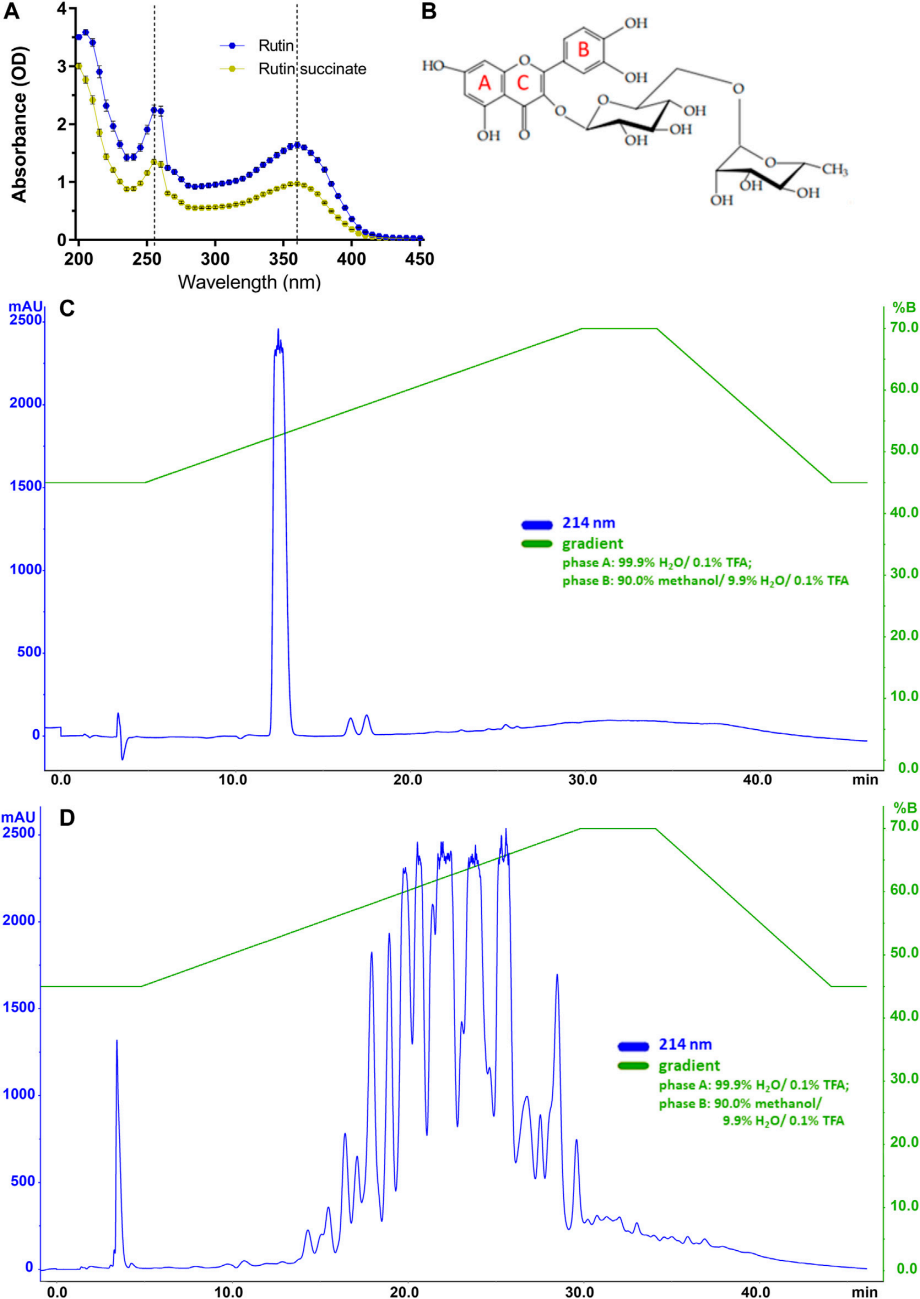
**(A)** The absorbance spectra of rutin and RS. **(B)** Molecular structure of rutin with indications of aromatic rings A, B and C; Chromatographic profiles obtained for **(C)** rutin and **(D)** RS analyzed by HPLC using a C18 column (250 × 4.6 mm). Chromatographic conditions: ACE C18 column (250 × 4.6 mm); mobile phase A: 99.9% H_2_O/0.1% TFA and B: 90.0% methanol/9.9% H_2_O/0.1% TFA); flow rate of 1.0 ml/min; λ = 214 nm; and injection volume of 20 μl.

RS showed a yellow coloration and a powdered aspect, as rutin; however, RS is more hygroscopic than rutin, and then it was maintained in the desiccator at room temperature. RS solubility in water is 115 times higher than that of rutin (14.4 g/L vs. 0.125 g/L, respectively). In the following experiments, RS molecular weight was considered as if it was the same of rutin (610.52). Regarding the absorbance spectrum, both rutin and RS showed peaks at 260 and 360 nm (Figures 2A,B), as expected for the aromatic rings A and B of flavonoids (Bondarev and Knyukshto, 2013; Kumar and Pandey, 2013).

Rutin and RS showed different analytical profiles when analyzed by HPLC (Figures 2C,D). Rutin presented a major peak with retention time (RT) at 13 min and absorption in 214, 154, and 280 nm. It is important to observe that this peak attributed to rutin was not present in the RS chromatographic profile, which displayed several peaks also with absorption in 214, 154, and 280 nm. These peaks in RS chromatogram are likely due to the different extent of succinylation of the hydroxyl groups present in rutinoside moieties of rutin. The presence of chemical residuals in RS synthesis was also analyzed by HPLC. Retention time for the peaks of chemicals used in synthesis (succinic acid: 6 min, succinic anhydride: 12 min, pyridine: 5 min, diethyl ether: 23 and 33 min) were not observed in RS samples, and therefore, RS synthesis and purification were considered successful and without chemical contaminants. Further purification of rutin peaks was not carried out, and the RS samples used in the experiments is therefore a combination of different succinylation forms of rutin.

**FIGURE 3:**
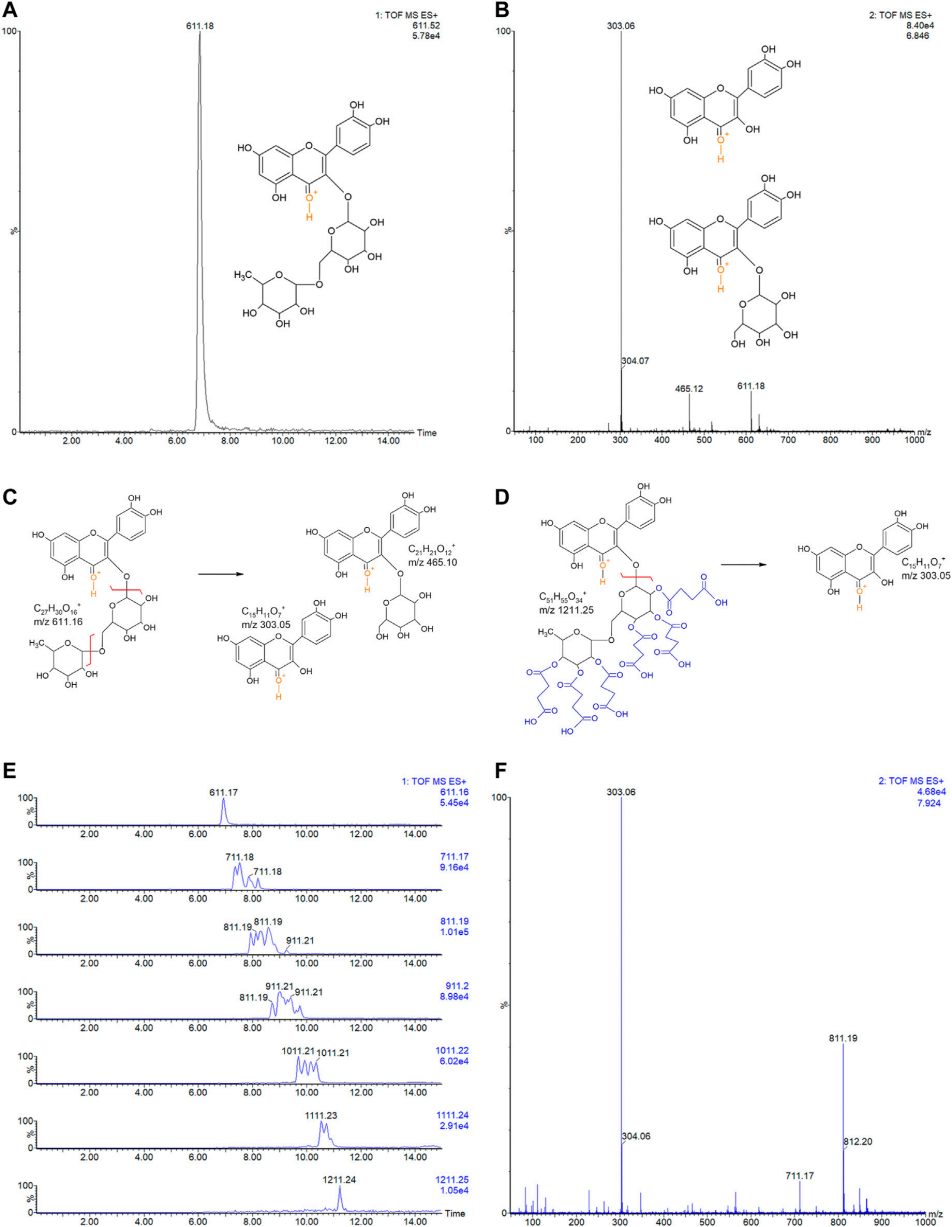
**(A)** LC-MSE chromatogram of rutin (10 ng, RT 6.84). **(B)** CID-MS/MS spectrum of rutin: main fragment ions observed at m/z 303.06+ and 465.12+. Remaining precursor ion at 611.18+ due to the DIA analysis. Proposed fragmentation routes of **(C)** rutin and **(D)** RS by ESI-CID-MS/MS. **(E)** LC-MSE chromatograms of RS (500 ng) with multiple succinylations (1–6), and **(F)** representative CID-MS/MS spectrum of RS with 2 and 3 succinylations, at RT 7.924. Remaining precursor ions (at m/z 711.17+ and m/z 811.19+) due to the DIA analysis.

Rutin was analyzed by LC-MSE and eluted at 6.84 min, showing the expected precursor ion at m/z 611.18+ and main fragments at 303.06+ and 465.12+ (Figures 3A,B). Rutin has six possible succinylation sites on the free hydroxyl groups of its rutinoside (Figures 3C,D).

Ion chromatograms of RS showed that several succinylated forms co-exist, composing a complex mixture of 1–6 succinyl substitutions (Figure 3E). Six possible forms exist for 1 succinyl substitution on rutin and 15, 20, 15, and 6 forms exist for 2, 3, 4, and 5 succinyl substitutions, respectively. Indeed, the 2–5 RS shows the isomeric forms in the chromatograms of their precursor ions (Figure 3F). Each succinyl substitution adds 100.016 Da to rutin, so that the precursor ions of RS for 1–6 substitutions are observed at m/z 711.2, 811.2, 911.2, 1011.2, 1111.2, and 1211.2, respectively. Only the original rutin and the 6-succinylated rutin show single forms and single-peak chromatograms (Figure 3E). Therefore, a total of 63 RS forms co-exist in the mixture, plus part of the remaining original non-substituted rutin. Low percentages of rutin and RS with 5 and 6 succinates are present in the sample (3.9, 4.8, and 0.6%, respectively). Medium rates are observed for 1 and 4 succinate RS (17.3 and 15.3%); however, the RS sample is mostly composed by RS with 2 and 3 succinates (30.7 and 27.6% of the sample).

**FIGURE 4:**
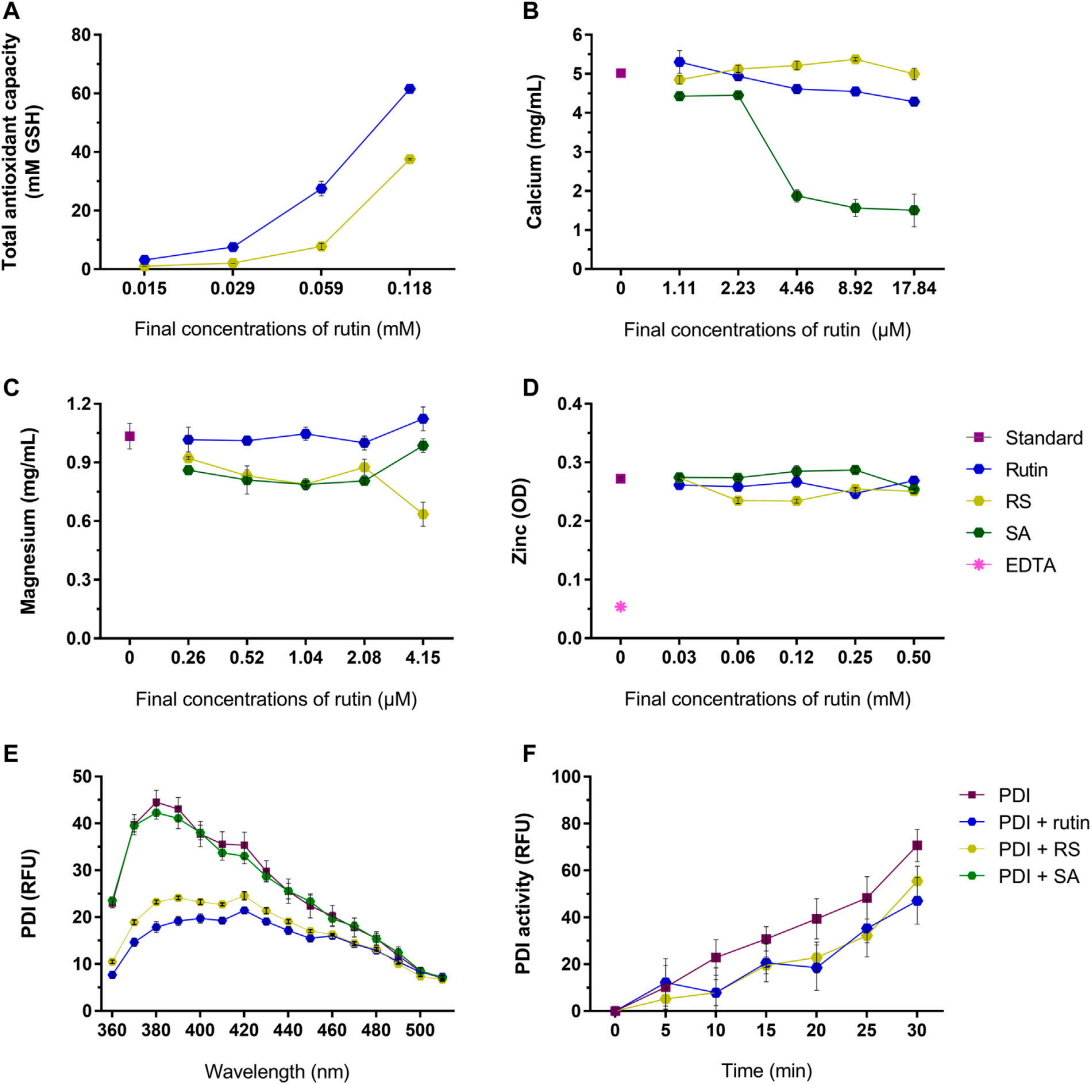
**(A)** Total antioxidant capacity of rutin and RS at different concentrations, analyzed by the CUPRAC method. Data were expressed as GSH equivalents (mM). Quenching of **(B)** calcium, **(C)** magnesium and **(D)** zinc by the pre-incubation with rutin, RS, SA or Na_2_EDTA at different concentrations. Data were expressed as concentration of free calcium or magnesium (mg/ml) or as optical density units (OD) for zinc. **(E)** Fluorescence spectra of PDI or PDI pre-incubated with rutin, RS or AS. Data were expressed as relative fluorescence units (RFU). **(F)** PDI reductase activity, evaluated by change in the fluorescence of di-eosine-GSSG probe (150 nM); PDI was tested alone or PDI pre-incubated with rutin, RS and SA (60 µM). Data were expressed as relative fluorescence units (RFU). Assays for **(A–D)** were carried out in duplicate and for **(E,F)** in triplicate.

Rutin has a broad range of activities, therefore it was relevant to investigate if RS also exhibit these activities, as antioxidant (Ganeshpurkar and Saluja, 2017), quencher of metal ions (Behling et al., 2004; Pietta, 2000; Kandaswami and Middleton, 1994; Afanas’ev et al., 1989; Ganeshpurkar and Saluja, 2017) and PDI inhibitor (Jasuja et al., 2012). Rutin and RS showed a concentration-dependent antioxidant activity (Figure 4A), but RS expressed a lower antioxidant potential. Differently, rutin and RS did not show expressive quenching of calcium (Figure 4B), magnesium (Figure 4C) or zinc (Figure 4D) ions; however succinic acid (SA, the free-form of succinic anhydride) quenched calcium ions in a concentration-dependent manner.

Rutin is a known PDI inhibitor (Jasuja et al., 2012), as it modulates PDI structure and function. Rutin and RS, but not SA, altered PDI chemical structure, decreasing the fluorescence of Trp residues of PDI (Figure 4E). Congruently, pre-incubating rutin or RS with PDI decreased its reductase activity, evidenced by the decrease in the RFU of the probe (Figure 4F), as well as the decrease in the Vmax of the reaction (PDI alone: 0.043 ± 0.0003 units/second, PDI + rutin: 0.030 ± 0.004 and PDI + RS: 0.03 3 ± 0.001 units/second). Therefore, both rutin and RS inhibited PDI reductase activity and acted as antioxidants, since RS presented the same core chemical structure of the flavonoid rutin (Pedriali et al., 2008b).

### RS Alters Hemostatic Parameters *In Vitro*

Besides rutin activity as a PDI inhibitor, studies have already shown that rutin may modulate other hemostatic components *in vitro* (Kuntić et al., 2011; Choi et al., 2015). Therefore, we investigated the direct activity of rutin and RS in hemostatic parameters *in vitro*, as well as on abundant proteins present in blood, as fibrinogen and albumin. To better understand RS activities, SA was also analyzed comparatively, since RS was synthetized by succinylation of rutin.

**FIGURE 5:**
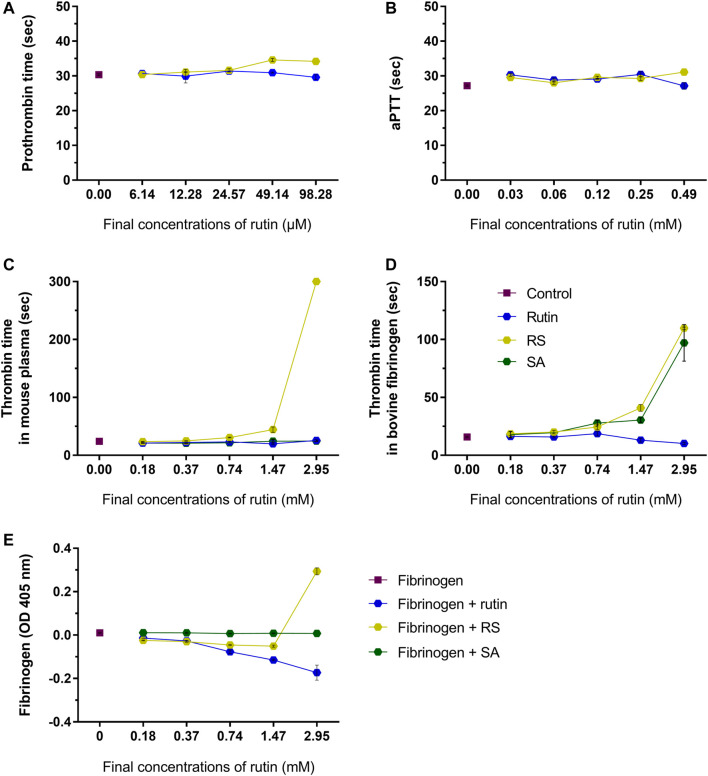
Activity of rutin and RS at different concentrations on **(A)** prothrombin time in mouse plasma; **(B)** aPTT in mouse plasma. Activity of rutin, RS and SA at different concentrations on **(C)** thrombin time in mouse plasma; **(D)** thrombin time in bovine fibrinogen; **(E)** formation of complexes with fibrinogen. Data are representative of assays in triplicate.

RS prolongated moderately prothrombin time (Figure 5A) and aPTT (Figure 5B) (maximum of 3.6 and 3.9 s compared to control, respectively), which indicated that RS could affect some component(s) of the common pathway of the coagulation cascade. Later on, thrombin time was analyzed using mouse plasma (Figure 5C) or bovine fibrinogen (Figure 5D) as substrates. Compared to control, the thrombin time was prolonged when RS was added (maximum of >300 s for mouse plasma and 93.7 s for fibrinogen). SA prolonged the thrombin time when fibrinogen solution was used as the substrate (increase of 81 s), whereas rutin shortened the thrombin time in 6.5 s.

Since fibrinogen is a key component of hemostasis, the influence of rutin, RS and SA was investigated in this protein. The analysis of possible complexes with fibrinogen (Figure 5E) revealed that only the highest concentration of RS induced an increase in fibrinogen turbidity, which indicates a possible formation of complex by electrostatic interaction (Zhang et al., 2013). SA did not form complexes with fibrinogen, and the inhibition of thrombin time by SA may be more related with its activity as a calcium quenching molecule, as demonstrated above. Since only RS altered all hemostatic parameters, its activity on coagulation is different from that shown by rutin, and not due solely to the presence of succinates in its structure.

**FIGURE 6:**
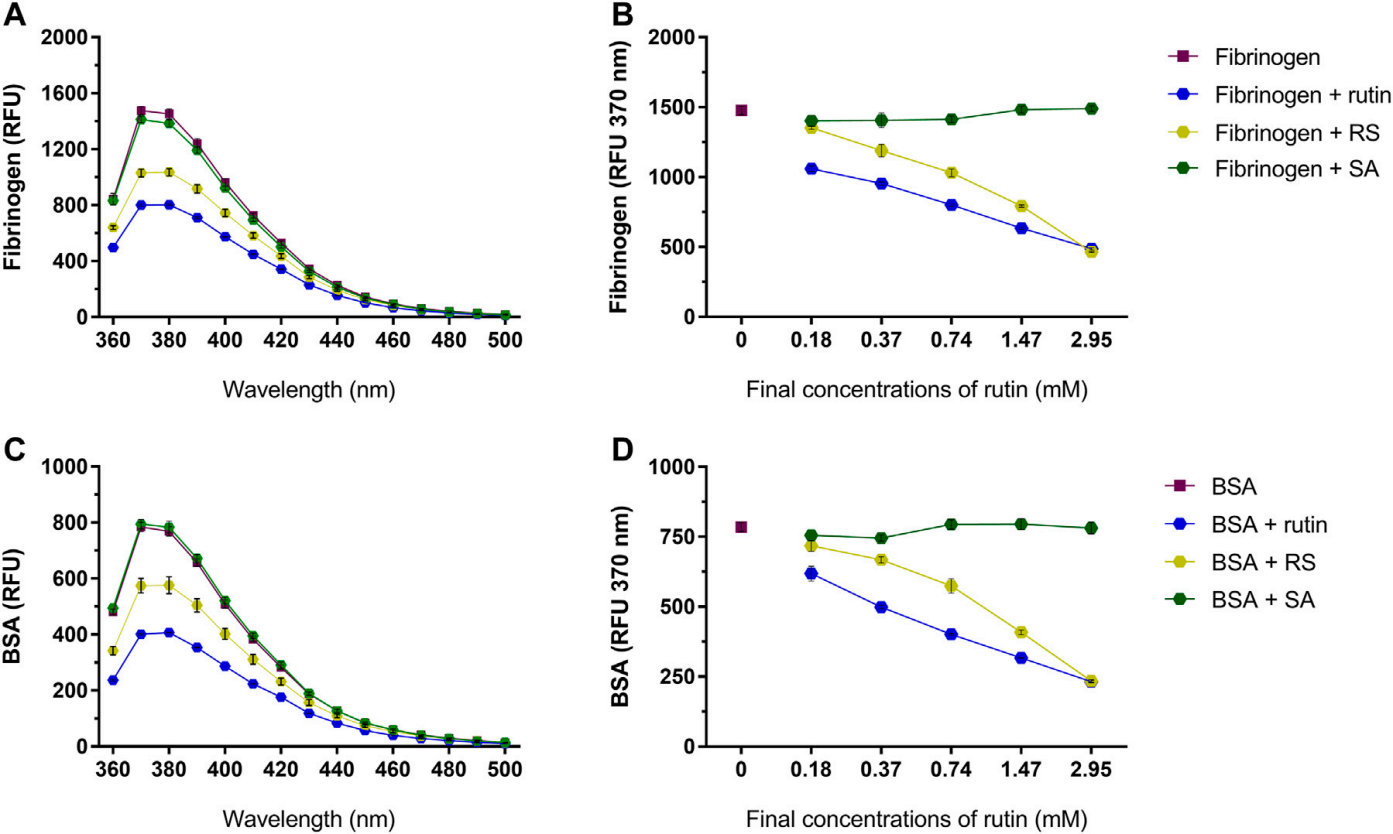
Activity of rutin, RS and SA at different concentrations on the fluorescence spectra of **(A,B)** bovine fibrinogen and **(C,D)** BSA. Data are expressed as RFU, and assays were carried out in triplicate.

Pre-incubation of rutin and RS with fibrinogen (Figures 6A,B) or albumin (Figures 6C,D) decreased the fluorescence of their Trp residues, indicating a possible alteration of their 3D structure, which was not observed by pre-incubation with SA.

### RS Inhibits BjV Activities *In Vitro*

**FIGURE 7:**
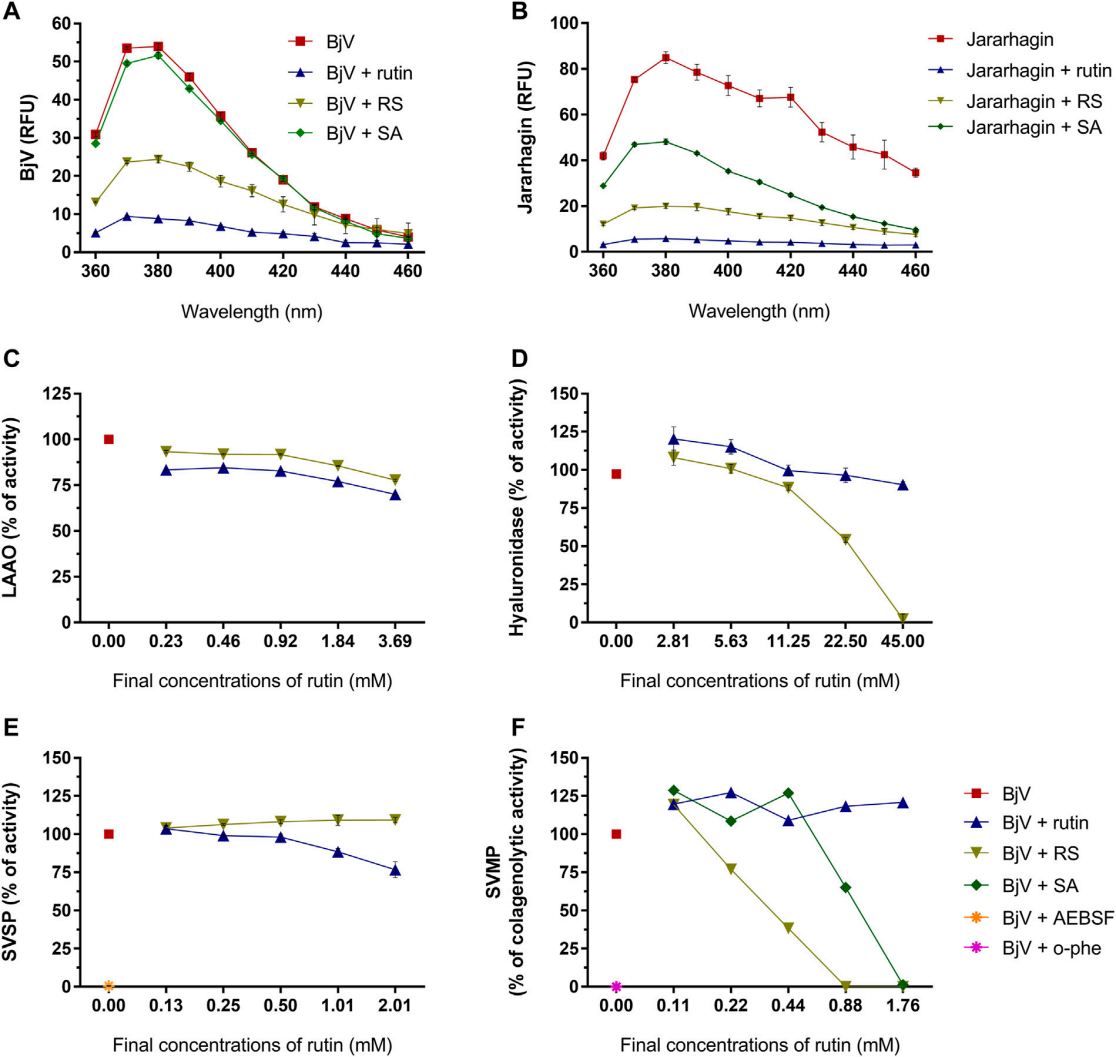
Activity of rutin, RS and SA on the fluorescence spectra of **(A)** BjV and **(B)** jararhagin. Data were expressed as RFU. Activity of rutin, RS, SA, AEBSF (SVSP inhibitor) and o-phe (SVMP inhibitor) on enzymatic activities of BjV protein families *in vitro*: **(C)** LAAO, **(D)** hyaluronidase, **(E)** SVSP and **(F)** SVMP. SA and o-phe were tested only for SVMP activity, and AEBSF was tested only for SVSP activity. Data were expressed as percentage of the maximum enzymatic activity; assays were carried out in duplicates/triplicates.

As the focus of this work is the use of rutin and RS as therapeutic compounds for the treatment of snakebites, the possible modulation of BjV biological activities by rutin and RS was first assessed *in vitro*. Secondly, the direct activity of rutin, RS and SA was also tested in jararhagin – the most abundant SVMP in BjV (Sugiki et al., 1995). As observed for fibrinogen, albumin and PDI, rutin and RS decreased the fluorescence of Trp residues in BjV proteins (Figure 7A) and jararhagin (Figure 7B). However, SA only modulated the fluorescence of jararhagin. In order to verify whether protein conformational alterations were also modifying BjV activities, *in vitro* tests were undertaken.

Rutin and RS minimally inhibited LAAO activity (max. of 30.1 and 22.3% of inhibition, respectively) (Figure 7C). However, differently from rutin, RS inhibited BjV hyaluronidase activity (Figure 7D) in a concentration-dependent manner, showing a maximum of 97.7% of inhibition. LAAO and hyaluronidase are minor parts of BjV, and therefore, other major protein families of BjV were chosen to be tested, as SVSP and SVMP.

SVSP activity (Figure 7E) was completely inhibited (99.4%) by AEBSF, an SVSP inhibitor, and modestly inhibited by the pre-incubation with rutin (max. of 23.3% of inhibition), but not by RS. On the other hand, rutin did not alter the collagenolytic activity of SVMP (Figure 7F), while BjV pre-incubated with o-phe, an SVMP inhibitor totally blocked this activity. Similarly, pre-incubation of BjV with RS or SA inhibited the collagenolytic activity in a concentration-dependent manner, achieving 100% of inhibition on the highest concentrations tested. To confirm RS and SA ability to inhibit SVMP, other SVMP activities were tested.

**FIGURE 8:**
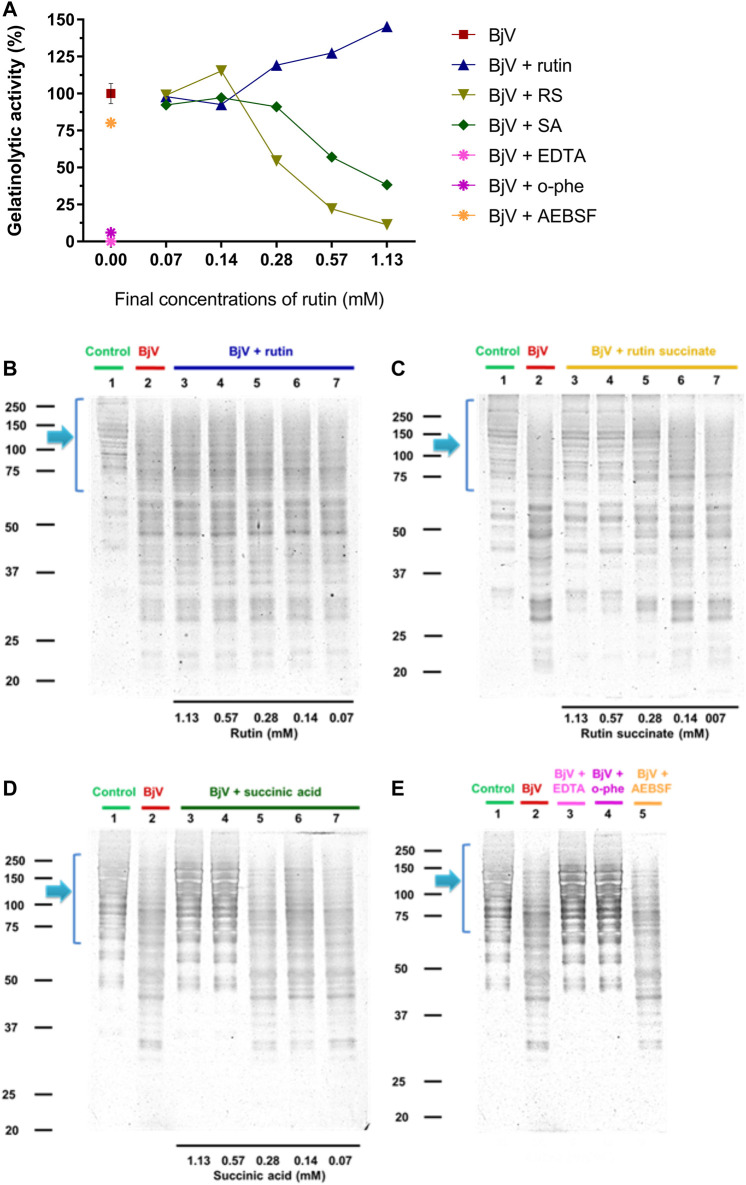
**(A–D)** Activity of rutin, RS and SA at different concentration on BjV-induced gelatinolytic activity *in vitro*. **(A)** data were expressed as percentage of gelatinolytic activity induced by BjV alone; **(B–E)** gelatin degradation by BjV evaluated by means of SDS-PAGE gels (10%). Gelatin incubated without BjV (lanes 1) or with BjV solutions at 1.0 mg/ml (lanes 2); [**(B)**, BjV + rutin], lanes 3–7, rutin concentrations ranging from 1.13 to 0.07 mM; [**(C)**, BjV + RS], lanes 3–7, RS concentrations ranging from 1.13 to 0.07 mM; [**(D)**, BjV + SA], lanes 3–7, SA concentrations ranging from 1.13 to 0.07 mM; **(E)** BjV + Na_2_EDTA (lane 3), BjV + o-phe (lane 4) and BjV + AEBSF (lane 5). Arrows refer to interest bands of 75 kDa–250 kDa, relative to non-degraded gelatin.

**FIGURE 9:**
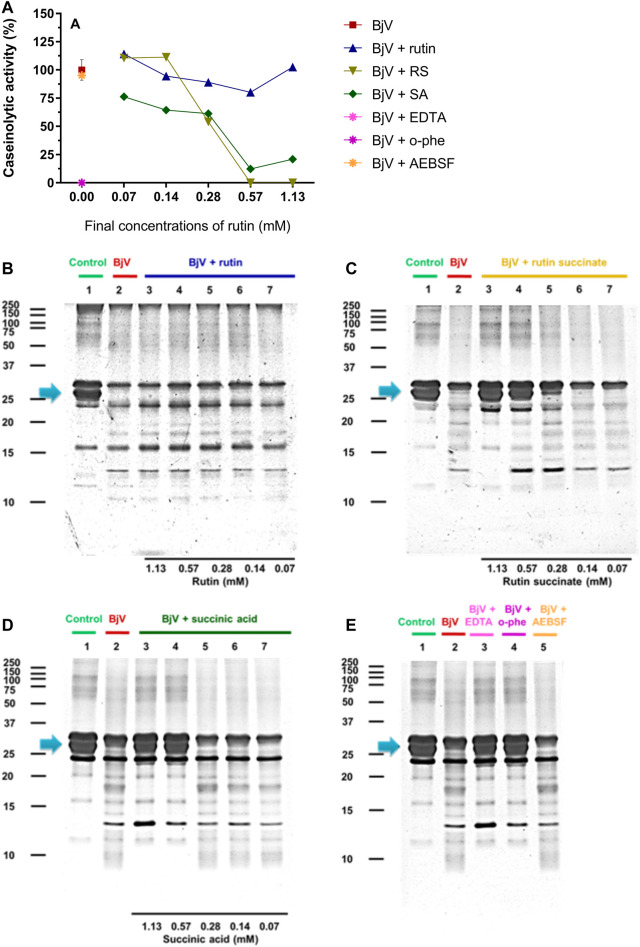
**(A–D)** Activity of rutin, RS and SA at different concentration on BjV-induced caseinolytic activity *in vitro*. **(A)** data were expressed as percentage of caseinolytic activity induced by BjV alone; **(B–E)** casein degradation by BjV evaluated by means of SDS-PAGE gels (10%). Casein incubated without BjV (lanes 1) or with BjV solutions at 1.0 mg/ml (lanes 2); [**(B)**, BjV + rutin], lanes 3–7, rutin concentrations ranging from 1.13 to 0.07 mM; [**(C)**, BjV + RS], lanes 3–7, RS concentrations ranging from 1.13 to 0.07 mM; [**(D)**, BjV + SA], lanes 3–7, SA concentrations ranging from 1.13 to 0.07 mM; **(E)** BjV + Na_2_EDTA (lane 3), BjV + o-phe (lane 4) and BjV + AEBSF (lane 5). Arrows refer to interest bands of 25 kDa–30 kDa (approximately), relative to non-degraded α-casein.

**FIGURE 10:**
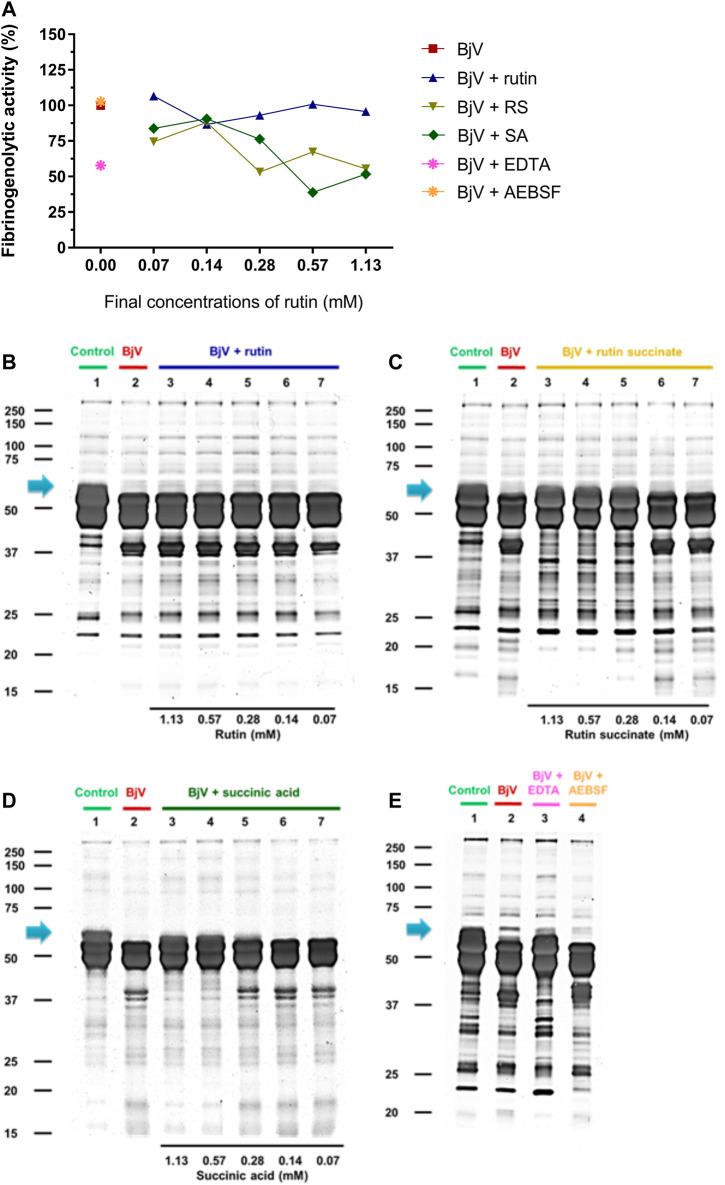
**(A–D)** Activity of rutin, RS and SA at different concentration on BjV-induced fibrinogenolytic activity *in vitro*. **(A)** Data were expressed as percentage of fibrinogenolytic activity induced by BjV alone; **(B–E)** fibrinogen degradation by BjV was evaluated by means of SDS-PAGE gels (10%). Fibrinogen incubated without BjV (lanes 1) or with BjV solutions at 1.0 mg/ml (lanes 2); [**(B)**, BjV + rutin], lanes 3–7, rutin concentrations ranging from 1.13 to 0.07 mM; [**(C)**, BjV + RS], lanes 3–7, RS concentrations ranging from 1.13 to 0.07 mM; [**(D)**, BjV + SA], lanes 3–7, SA concentrations ranging from 1.13 to 0.07 mM; **(E)** BjV + Na_2_EDTA (lane 3), BjV + o-phe (lane 4) and BjV + AEBSF (lane 5). Arrows refers to interest bands of 60 kDa (approximately), relative to non-degraded fibrinogen α-chain.

The proteolytic activity of BjV was analyzed on the substrates gelatin, casein and bovine fibrinogen (Figures 8–10, lanes 2). BjV alone cleaved protein bands of 75 kDa–250 kDa bands for gelatin, 25 kDa–30 kDa bands for casein (α-casein) and 60 kDa bands for fibrinogen (α-chain), and subsequent increases in lower molecular weight bands, related to products of protein degradation of substrates. BjV pre-incubated with AEBSF (Figures 8A–10A,E) or rutin (Figures 8A–10A,B, lanes 3–7) minimally attenuated proteolysis (maximum of 19.8% of inhibition) of those substrates. As expected, the inhibition of SVMP by Na_2_EDTA or o-phe decreased the BjV proteolytic activity (Figures 8A–10A,E): 100% for caseinolytic effect, 93.4% for gelatinolytic, and 42.2% for fibrinogenolytic activity. Likewise, RS inhibited SVMP activity more intensely than Na_2_EDTA or o-phe (Figures 8A–10A,C, lanes 3–7), in a concentration-dependent manner, and inhibition rates as high as 100% was noticed for caseinolytic activity, around 90% for gelatinolytic and 47% for fibrinogenolytic activity. As observed for the collagenolytic activity, SA exhibited lower rates of proteolysis inhibition than RS (Figures 8A–10A,D, lanes 3–7), and maximum decreases of 61.2%–79.1% were observed for the three substrates. In addition, RS and SA decreased the degradation of fibrinogen by jararhagin and the degradation of gelatin by the collagenase from *C. histolyticum*, indicating a broader modulation of different agents and toxins.

**FIGURE 11:**
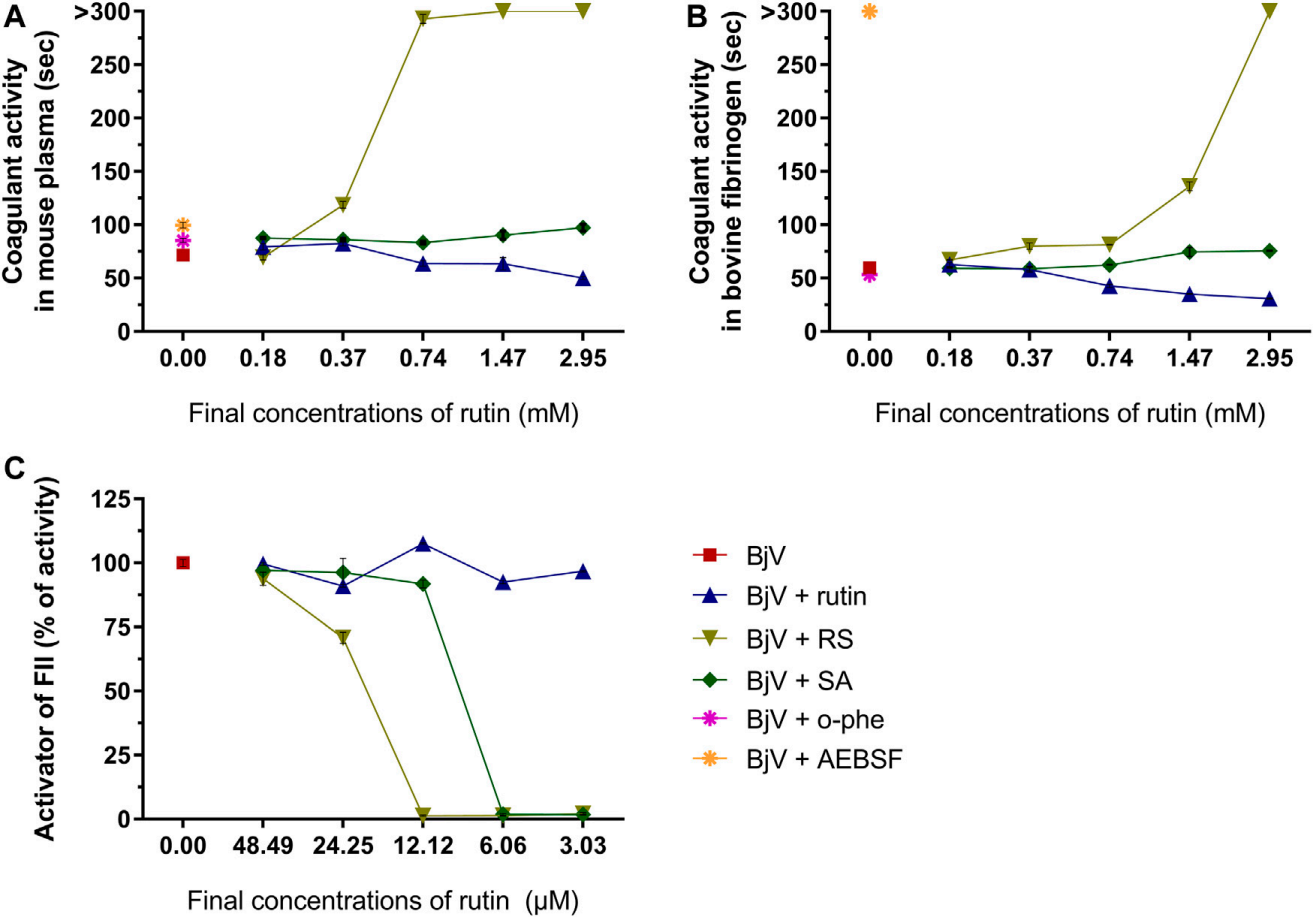
Activity of rutin, RS or AS at different concentrations on BjV activities *in vitro*: **(A)** clotting of mouse plasma or **(B)** bovine fibrinogen. Data were expressed as clotting activity in seconds after the addition of BjV (0.25 mg/ml). **(C)** Prothrombin activation. Data were expressed as percentage of activity, and assays were carried out in triplicates.

Besides the proteolysis of proteins, SVMP also showed clotting activity due to the activation of coagulation factors, as factor X and prothrombin (Sano-Martins et al., 2003; Serrano et al., 2014). Rutin interfered minimally (9% inhibition) with the activation of prothrombin by BjV (Figure 11C). However, the pre-incubation of BjV with RS and SA at the 2 higher concentrations tested decreased 98%–99% the activation of prothrombin induced by BjV. Since the *in vitro* clotting activity is a well-established characteristic of BjV, this property was tested for rutin, RS or SA using bovine fibrinogen (Figure 11B) or mouse plasma (Figure 11A) as substrates. The clotting time of fibrinogen or mouse plasma was minimally decreased by rutin (5.1% maximum inhibition), SA (21% maximum inhibition) or o-phe (16.6% maximum inhibition); however, SVSP inhibition by AEBSF inhibited completely fibrinogen clotting by BjV. Differently, RS decreased BjV clotting activity in a concentration-dependent manner, ranging from 11.3 to 100% inhibition for fibrinogen clotting, and 30%–100% inhibition for plasma clotting.

Therefore, rutin minimally interfered with BjV activities *in vitro.* Nonetheless*,* pre-incubation of RS or SA with BjV inhibited important activities thereof, mainly those related to SVMP activity. In addition, RS also displayed broader effects related to a direct modulation of coagulation, evidencing its potential as an anticoagulant compound.

Since rutin showed potential beneficial effects on envenomation *in vivo* (Sachetto et al., 2018) and RS directly inhibited BjV *in vitro*, both rutin and RS were tested using an experimental model of envenomation. For *in vivo* experiments, rutin and RS were tested in concentrations 9 times higher than those used for BjV, and therefore, at this dosage, rutin pre-incubated with BjV did not alter its proteins, while pre-incubation with RS (or SA) inhibited partially the SVMP biological activities.

### Rutin and RS Suppressed BjV Toxicity and Lethality

To analyze mouse survival as well as hemostatic disturbance, BjV was intraperitoneally injected in mice. Two different doses of BjV were used: 2×LD_50_ was used to simulate moderate envenomation and evaluate hemostatic parameters, whereas 3×LD_50_ was used to evaluate severe envenomation. Both rutin and RS were preincubated with BjV, and tested regarding its protective potential *in vivo*.

**FIGURE 12:**
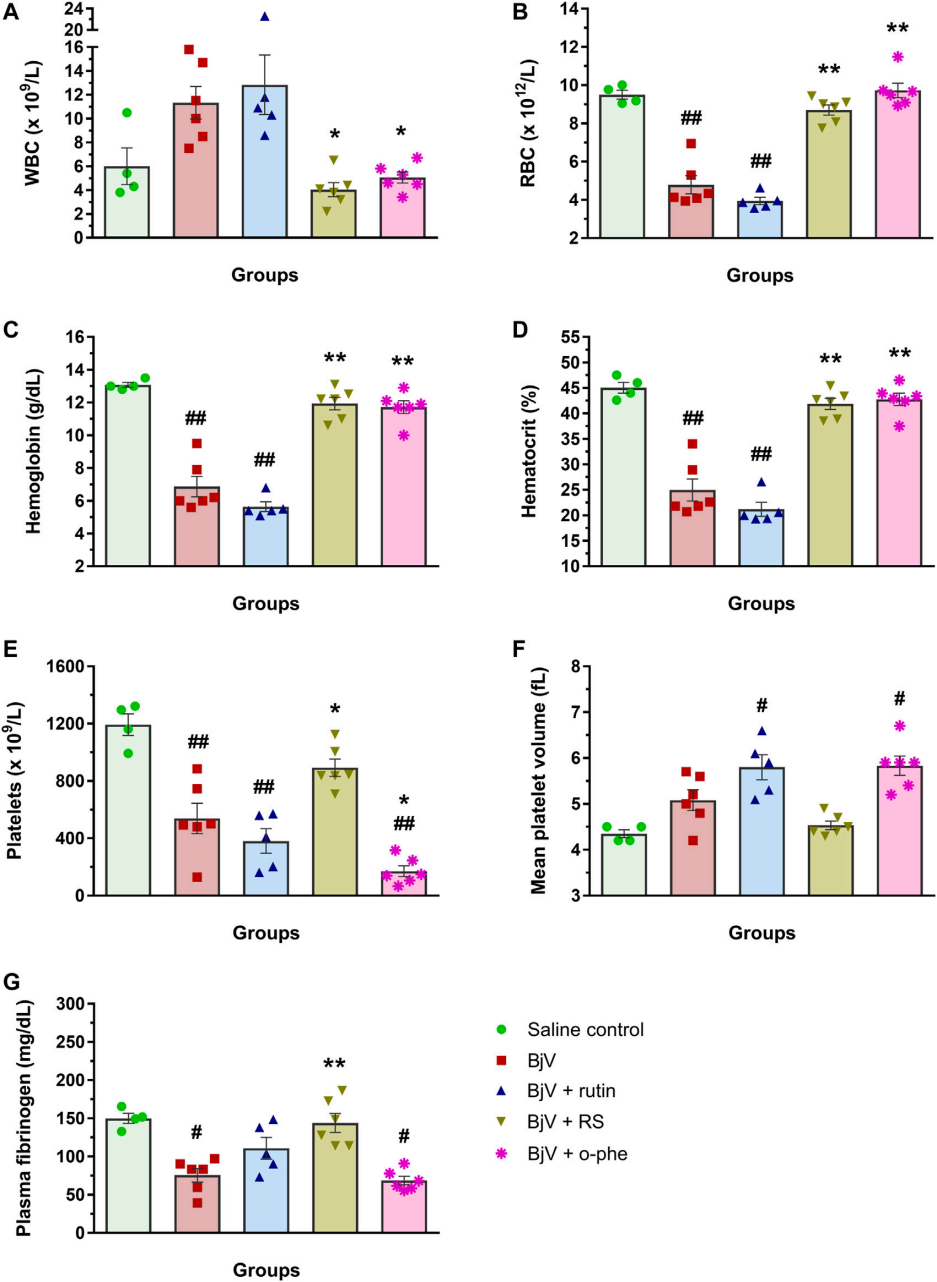
**(A)** WBC counts, **(B)** RBC counts, **(C)** hemoglobin, **(D)** hematocrit, **(E)** platelet counts, **(F)** MPV and **(G)** plasma fibrinogen in mice 48 h after the injection of saline, BjV, BjV + rutin, BjV + RS or BjV + o-phe (BjV dose: 2×LD_50_). One-way ANOVA was used, followed by Bonferroni *post-hoc* test; #*p* < 0.05 and ##*p* < 0.001 when compared to saline control group; *p < 0.05 and **p < 0.001 when compared to BjV group. Data are expressed as mean ± SEM. (*n* = 4–6/group).

The moderate envenomation model did not induce mortality in mice in 48 h. The hematologic analysis revealed that preincubating BjV with RS or o-phe decreased circulating WBC (Figure 12A) compared to BjV group (*p* < 0.050). BjV alone induced a characteristic decrease of 44%–58% in RBC parameters (Figures 12B–D) (*p* < 0.001), but BjV + RS and BjV + o-phe hindered such fall, and results similar to the control (*p* = 1.000) and different from BjV (*p* < 0.001) were noticed. BjV also evokes other hemostatic disturbances *in vivo*, as thrombocytopenia and hypofibrinogenemia. The experimental groups BjV, BjV + rutin and BjV + o-phe had 53%–86% lower platelet counts and 16%–33% higher mean platelet volume (MPV, *p* < 0.050, Figure 12F) than the saline control (*p* < 0.001, Figure 12E). BjV and BjV + o-phe groups also showed a marked decrease in plasma fibrinogen levels (*p* < 0.050, Figure 12G), but rutin partially prevented the drop in fibrinogen levels, not differing from control (*p* = 0.201). As observed for the RBC, platelet counts and fibrinogen levels in the BjV + RS group were similar to the those of the control group (*p* = 1.000), and higher than those of mice injected with BjV alone (*p* < 0.050 for platelets and *p* < 0.0001 for fibrinogen). In addition to the hemostatic disturbances described above, BjV also evoked hemorrhages in the abdominal wall of all envenomed mice. However, the pre-incubation of BjV with RS or o-phe completely prevented it.

**FIGURE 13:**
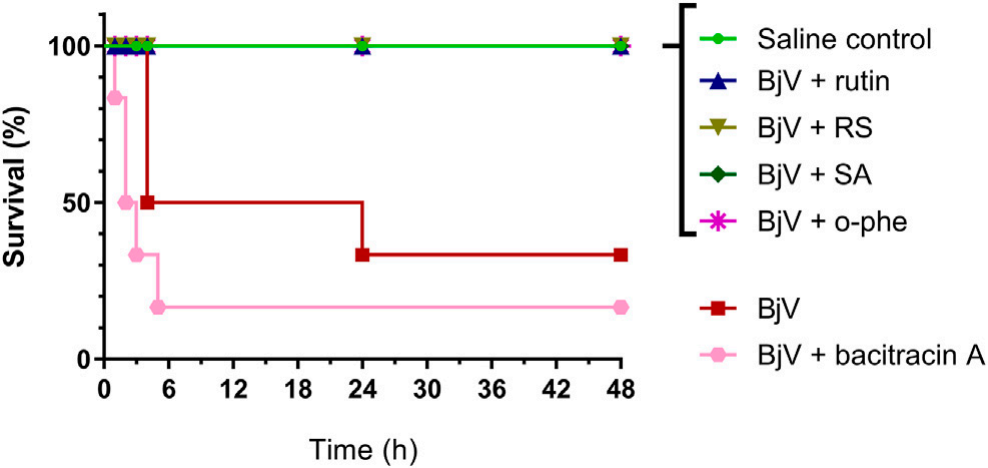
Survival curves of mice during 48 h after the injection of saline, BjV, BjV + rutin, BjV + RS, BjV + SA, BjV + o-phe and BjV + bacitracin A (BjV dose: 3×LD_50_). Log-rank test was used, and survival curves showed significant difference (*p* = 0.005). Data were expressed as percentage of survival (*n* = 3–6/group).

The severe envenomation model allowed the observation of mice survival for 48 h after 3×LD_50_ BjV injection (Figure 13). All animals from saline control, BjV + rutin, BjV + SA and BjV + o-phe groups survived. However, when injected with BjV alone, 50% of animals died after 4 h and the final survival rate at 48 h was 33.3%. Animals injected with BjV and bacitracin A (a thiol isomerase inhibitor) showed even lower survival rates (16.7%) after 48 h. Survival curves (Figure 13) showed significant differences among groups (*p* < 0.001). The results indicated that rutin, RS, AS and o-phe inhibited the toxic activity of BjV, preserving mice survival in a severe model of systemic envenomation. Furthermore, the thiol isomerase inhibition by bacitracin A reduced mice survival, which indicate a protective role of these enzymes in the organism during envenomation.

**FIGURE 14:**
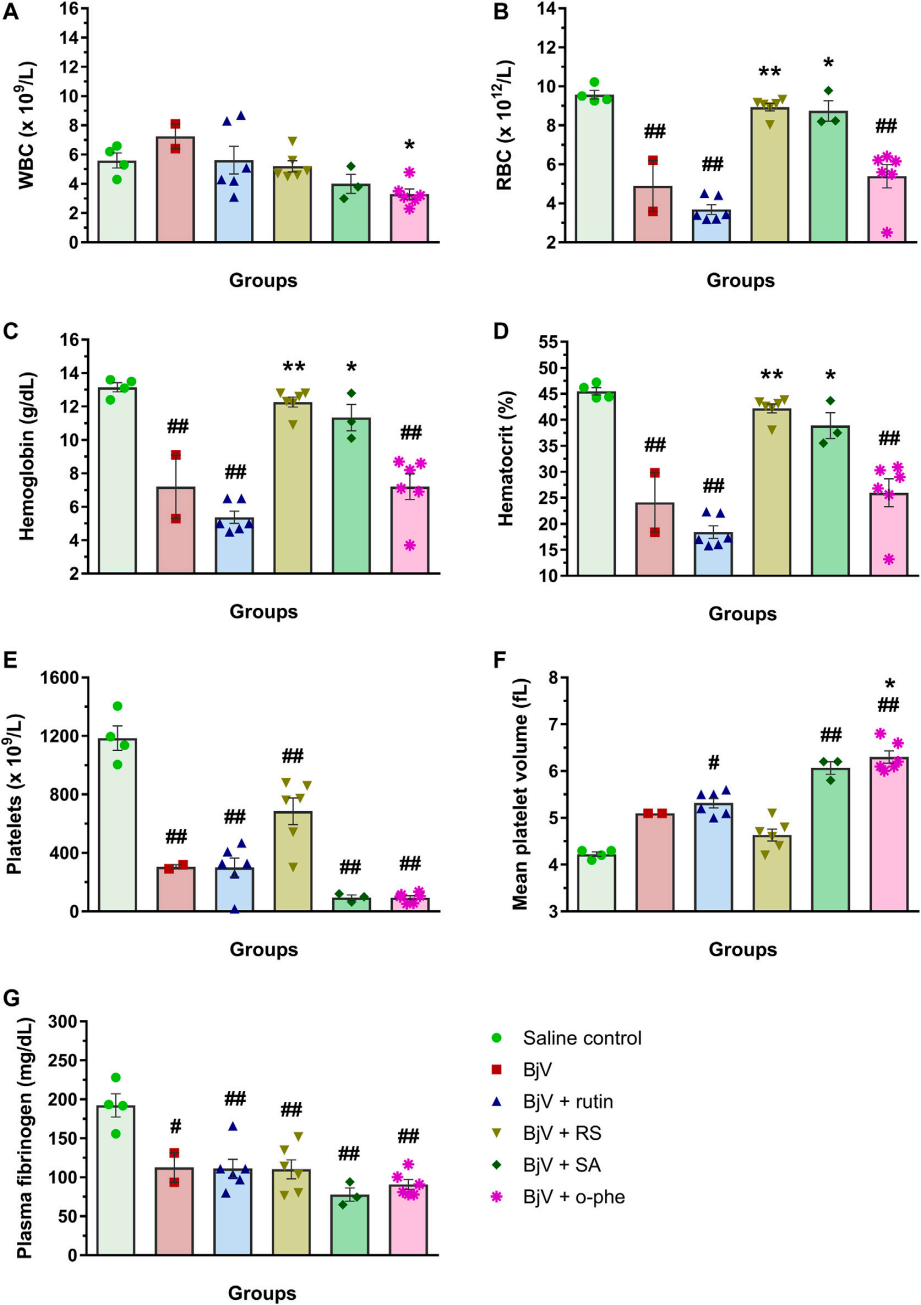
**(A)** WBC counts, **(B)** RBC counts, **(C)** hemoglobin, **(D)** hematocrit, **(E)** platelet counts, **(F)** MPV and **(G)** plasma fibrinogen of mice 48 h after the injection of saline, BjV, BjV + rutin, BjV + RS, BjV + SA and BjV + o-phe (BjV dose: 3LD_50_). One-way ANOVA was used, followed by Bonferroni *post-hoc* test; #*p* < 0.05 and ##*p* < 0.001 when compared to saline control group; *p < 0.05 and **p < 0.001 when compared to BjV group. Data are expressed as mean ± SEM. (*n* = 3–6/group).

After 48 h of venom administration, hemostatic parameters of surviving mice were analyzed. WBC counts (Figure 14A) showed no alterations among groups (*p* > 0.129); nevertheless, RBC parameters (Figures 14B–D) showed results similar to those observed in the moderate envenomation model. BjV decreased RBC counts, hemoglobin and hematocrit (*p* < 0.001 compared to control), but the group BjV + RS and BjV + SA were similar to the control saline (*p* = 1.000) and different from the BjV group (*p* < 0.050). However, contrary to the moderate envenomation, BjV + o-phe group still showed a decrease of 43%–45% in RBC parameters compared to the control (*p* < 0.001). The analysis of blood smears revealed that envenomed mice presented morphological alterations as the presence of anisocytosis, polychromasia, schizocytes, dacryocytes and acanthocytes. This indicated that SVMP inhibition and rutin did not prevent RBC drop in the severe envenomation. However, RS and SA could prevent it, indicating the effectiveness of succinate groups on this parameter, regardless of SVMP inhibition. A marked thrombocytopenia was observed in all envenomed mice (Figure 14E) compared to controls (*p* < 0.001), as well as an increase in MPV (Figure 14F) in BjV + rutin, BjV + SA and BjV + o-phe groups (*p* < 0.050), but BjV + RS group showed the less intense decrease in platelet counts (42%). Plasma fibrinogen levels (Figure 14G) also decreased around 38%–60% in all envenomed animals (*p* < 0.050), and results showed that fibrinogen levels of BjV + rutin mice were similar in both moderate and severe envenomation.

Like the moderate envenomation model, severe envenomation induced hemorrhages in the abdominal and diaphragm of the animals, except in mice from the saline control, BjV + RS, BjV + SA and BjV + o-phe. Interestingly, BjV + rutin mice did not developed diaphragm hemorrhages neither. In one mouse that received BjV alone and died before 48 h, organs were collected for histological analyses. Skeletal muscles in the abdominal wall, at the face that had peritoneum intermediating their direct contact with BjV, showed paler eosinophilic coloration in their cytoplasm, implying that they had been damaged. Areas of hemorrhage were also observed at this face, but inflammatory infiltrate was not extensive. Muscle fibers present in the diaphragm showed the same lesions. In mice that received BjV preincubated with rutin and that survived for 48 h, a decreased extension of muscle fibers, in the abdominal wall and diaphragm showed decreased coloration of cytoplasm, and hemorrhage was less intense. In the mouse that received BjV preincubated with RS, no changes were observed. Thereby, the results of *in vivo* envenomation showed that BjV induced systemic hematological and hemostatic alterations and evoked animal lethality. SVMP inhibition (by o-phe and SA) prevented the hemorrhagic and lethal activity of BjV, but did not show effects on other parameters. Whereas rutin induced a partial improvement of fibrinogen levels, RS improved hematological and hemostatic parameters and completely inhibited BjV-induced hemorrhagic activity. This indicates that RS effectiveness is not limited to its succinate moieties nor SVMP inhibitory activity. Moreover, rutin and RS ensured mice survival, showing their potential to neutralize the venom toxic activities, preventing the lethality evoked by BjV.

## Discussion

BjV is composed by a complex mixture of biomolecules, mostly proteins, as SVMP, SVSP, LAAO, hyaluronidases, among others (Koh et al., 2006; Deolindo et al., 2010; Sajevic et al., 2011; Takeda et al., 2012; Izidoro et al., 2014). These proteins present *in vitro* and *in vivo* activities, both in experimental models as well as in bitten patients, inducing hemostatic disturbances, bleeding and inflammatory reactions (Sano-Martins et al., 2003; Baldo et al., 2010; Gutiérrez, 2016; Kini and Koh, 2016; Gutiérrez et al., 2017). The severity of *Bothrops* envenomation is stratified as mild, moderate or severe, for proper patient treatment and antivenom administration (França et al., 2003). *B. jararaca* snakebite envenomation induces systemic alterations, as thrombocytopenia, a decrease in RBC counts and hypofibrinogenemia, both in animal models (Yamashita et al., 2014; Sachetto et al., 2018) and bitten patients (Maruyama et al., 1990; Santoro et al., 2008c). Thus, the analysis of hemostatic disturbances, such as blood incoagulability, hypofibrinogenemia, and other hematological alterations are relevant parameters to evaluate both the severity of envenomation and the effectiveness of antivenom administration (Santoro et al., 2008c). Therefore, the possible inhibitory potential of rutin and RS were tested regarding the characteristic enzymatic activities of BjV *in vitro*, as well as their potential to inhibit the toxicity and lethality of BjV in experimental envenomation models.

In order to achieve that, RS was synthetized. It showed higher water-solubility than rutin, in agreement with previous findings that reported an increase in 80 times in its solubility (Pedriali et al., 2008b). There was no alteration at the RS core chemical structure, as shown by the absorbance of their aromatic rings A and B (Bondarev and Knyukshto, 2013; Kumar and Pandey, 2013), and the CID-MS/MS fragments (Ou-yang et al., 2013; Chen et al., 2015), maintaining thereby the features of a flavonoid, like rutin. Furthermore, the differences between rutin and RS by HPLC and LC-MSE analyses were related to the substitutions of hydroxyl groups in the sugar moieties of rutin for succinate groups, as shown previously (Pedriali, 2006), which indicated the effectiveness of the rutin succinylation process. Rutin succinylation did not alter the characteristic activities of rutin, however the observed difference between rutin and RS antioxidant capacity may be due to the normal range of antioxidant activity of the flavonoids, which differs even within quercetin molecules. Besides, the results are in accordance with previous reports in which it was shown that RS has a lower antioxidant activity due to its lack of stabilization of carboxyl groups by hydroxyl groups in RS (Pedriali, 2006; Pedriali et al., 2008b). Differently from rutin and RS, succinic acid presented a calcium quenching activity, which may be related to its ability of interacting and forming complexes with metal ions (Domingo et al., 1988; Pathan et al., 2014).

Another key feature of rutin is its ability to inhibit PDI. It is known that PDI is susceptible to alterations of structure and function depending on the redox state and its interaction with substrates (Bekendam et al., 2016). PDI possesses 5 Trp residues, and it was already observed that GSSG – a known substrate that interacts with PDI (Raturi and Mutus, 2007) – induces a decrease in fluorescence of Trp residues in PDI (Ado et al., 2006), as observed herein. Congruently, rutin binds to the hydrophobic pocked of PDI b’ domain, which induces a conformational alteration to a more compact structure, preventing other substrates to bind and inhibiting PDI reductase activity (Lin et al., 2015). It was already shown that only quercetins with glycosides at 3′ position in C ring are able to bind and inhibit PDI (Furie and Flaumenhaft, 2014), and in the present work it was observed that rutin and RS impacted in PDI structure, but not AS, which does not present the chemical structure and properties of rutin. Extracellular PDI is important for thrombus formation *in vivo* and rutin inhibits PDI both *in vitro* and *in vivo*. Rutin and analogous components inhibit thrombus formation in mice, as evaluated by the decrease in platelet accumulation and fibrin deposition (Jasuja et al., 2012). However, inhibition of the thiol isomerase family by bacitracin A was inefficient to protect mice from envenomation, therefore other activities of rutin – as anti-inflammatory, antioxidant, and modulator of vascular tonus and permeability – are likely involved therein (Afanas’ev et al., 1989; Ganeshpurkar and Saluja, 2017; Choi et al., 2015; Jasuja et al., 2012; Stopa et al., 2017; Schulman et al., 2016; Sharma et al., 2013; Panche et al., 2016; Kauss et al., 2008; Kirchner et al., 2013).

In addition, rutin was already shown to have the potential of interfering with other proteins in the blood, as showed by the results for BSA and fibrinogen. The activity of rutin on BSA was demonstrated earlier, and the decrease in albumin fluorescence was related with rutin binding by means of hydrogen bridges and weak van der Waals forces (Sengupta et al., 2018). The decrease in Trp residue fluorescence is also associated with conformational alterations of fibrinogen, since the Trp residues are mostly located in the hydrophobic portion of fibrinogen (Zhang et al., 2013). Our results confirm the interaction of rutin and RS with BSA and fibrinogen, which is important to understand their role *in vivo*, since BSA is relevant for the transport and bioavailability of exogenous components in the organism, whereas fibrinogen is a key component of hemostasis.

Regarding hemostasis components, SVSP and SVMP – the major protein families found in BjV – are both responsible for the clotting activity of BjV (Santoro and Sano-Martins, 1993; Yamashita et al., 2014; Sachetto et al., 2018), as also demonstrated herein. SVMP act as procoagulant proteins due to its ability to directly activate factor X and prothrombin (Antunes et al., 2010; Sachetto and Mackman, 2019), and were already related to the hemostatic disturbances evoked by BjV (Yamashita et al., 2014); (Senise et al., 2015). SVSP may act as thrombin-like enzymes, which are capable of inducing *in vitro* and *in vivo* coagulation (Santoro and Sano-Martins, 1993; Sano-Martins et al., 2003; Antunes et al., 2010; Yamashita et al., 2014), and have been pointed out as important toxins for venom-induced coagulopathy (Nahas et al., 1979; Stocker et al., 1982). Quercetins reversely inhibit serine proteases, as human thrombin and, possibly, thrombin-like enzymes (Mozzicafreddo et al., 2006; Cuccioloni et al., 2009; Xue et al., 2017). This effect is concentration-dependent, as observed for the inhibition of SVSP activity by rutin. Moreover, the different structures of flavonoids interfere on their potential to bind to serine proteases (Mozzicafreddo et al., 2006), and therefore it is possible that the succinate groups of RS favor the inhibition of BjV thrombin-like enzymes, preventing their clotting ability.

Rutin was previously studied when pre-incubated with BjV, and it did not show significant inhibitory activity on BjV activities *in vitro* (Sachetto et al., 2018), as also observed herein. However, the reduction in fluorescence of Trp residues has been considered an indicative of conformational alterations of proteins present in snake venoms (Hashkel et al., 2018), and quercetin has been already demonstrated to bind to *Bothrops* venom components and induce that change (Kumar et al., 2017). In fact, the modest inhibition of BjV toxins by rutin may be due to its indirect activities, as an antioxidant that neutralizes H_2_O_2_ generation by LAAO *in vitro*, or due to other effects that occurred only when high concentrations of rutin, as for SVSP inhibition, were used. However, RS displayed a higher ability to inhibit BjV toxins *in vitro*, as hyaluronidases and SVMP. Snake venom hyaluronidases are often related to the degradation of extracellular matrix components, mainly hyaluronic acid, contributing to local damage and venom propagation *in vivo*. Flavonoids are hyaluronidase inhibitors (Bala et al., 2018) and the marked effectiveness of RS compared to rutin may be due to chemical differences between them, and/or superior water solubility of RS.

The SVMP protein family is the most abundant in BjV and show several activities *in vitro* and *in vivo* (Markland and Swenson, 2013). As demonstrated previously (Sachetto et al., 2018), rutin did not modulate SVMP function, however the inhibition of SVMP proteolytic activity by RS and SA seemed to be directly related to the succinyl groups in these molecules. Degradation of protein components is an important activity of SVMP, mainly related to the hydrolysis of basal membrane components of capillary vessels, which is a fundamental step for the development of hemorrhages induced by venom *in vivo* (Gutiérrez et al., 2016a; Gutiérrez et al., 2016b). Studies have demonstrated the relevance of SVMP on systemic envenomation caused by *B. asper*, and that SVMP inhibitors decreased the clotting activity of venom *in vitro,* and defibrinogenation and hemorrhagic activities *in vivo* (Rucavado et al., 2004). As showed herein, RS effectively inhibited both hemorrhagic and defibrinogenating activities of BjV on the moderate model of envenomation. However, these inhibitors only partially prevented the increase in vascular permeability and mortality of envenomed animals (Rucavado et al., 2004; Chacón et al., 2015). The lethality of Viperidae snake venoms is attributed to intravascular consumption of coagulation factors induced by procoagulating venoms, and to blood leakage that leads to cardiovascular collapse by hemorrhagic venoms (Gutierrez et al., 2017). Studies suggest that *Bothrops* venoms lethality is multifactorial (Maria et al., 1998), which may comprise SVMP activities and the increase in inflammation and vascular permeability (Chacón et al., 2015). Our results demonstrate that BjV SVMP inhibition (by RS, SA or o-phe) is effective on preventing the hemorrhages and lethality induced by BjV. However, RS effects were certainly broader, and not only due to SVMP inhibition, as demonstrated by the beneficial effect of RS on blood cell counts during envenomation.

The decrease in RBC parameters by *Bothrops* envenomation has been already related to the occurrence of local and systemic bleedings and microangiopathic hemolytic anemia in animals (Senise et al., 2015; Sachetto et al., 2018)and patients (Santoro et al., 2008c; Málaque et al., 2019). Accordingly, our results showed that mice with lower RBC counts also manifested morphologic alterations of RBC – indicating that venom-induced intravascular hemolysis occurred (Senise et al., 2015) – and abdominal hemorrhage, suggesting a connection between bleedings and the decrease in RBC counts. The precise mechanisms of thrombocytopenia in *B. jararaca* envenomation are not completely elucidated yet, although it is known that it is not related to SVMP, SVSP or local hemorrhagic injury (Yamashita et al., 2014). Furthermore, more severely envenomed patients manifested a more prominent platelet decrease (Santoro et al., 2008c), as reported herein for mice. As mentioned above, blood incoagulability is attributed to SVMP and SVSP activities (Yamashita et al., 2014; Senise et al., 2015; Sachetto et al., 2018), and its association with thrombocytopenia increases the tendency to development of systemic bleedings (Kamiguti et al., 1991).

The use of plant compounds or extracts – including flavonoids – may inhibit totally or partially the lethality and hemorrhages induced by BjV (da Silva et al., 2015; de Souza et al., 2020; Domingos et al., 2015; Ferreira et al., 2019), however the compounds and mechanisms by which this inhibition occurs have not been described yet. Inasmuch as rutin and RS completely inhibited the lethality evoked by BjV and modulated BjV-induced hemostatic disturbances, it is likely that this effect is also due to a direct action in the organism, hindering the damaging consequences of envenomation.

It is important to notice that rutin is already commercialized as a food supplement in several countries and it is considered safe by the Food and Drug Administration (FDA) in recommended doses of 2 g per day for human beings. Chemical formulations containing rutin or analogous compounds (Varemoid®, Relvene®, Venoruton® e Paroven®) are commercialized since the 1960’s for the topical treatment of venous disturbances. Furthermore, quercetin and isoquercetin are being tested in clinical trials using oral administration (Heinz et al., 2010; Stopa et al., 2013; Flaumenhaft et al., 2015; Kooshyar et al., 2017; Di Lorenzo, 2018; Zwicker, 2018). Mice treated with rutin and other quercetins showed similar results on the inhibition of thrombus formation, either by oral or intravenous treatments (Jasuja et al., 2012). Reasoning that rutin and/or RS could be used as a first-choice medicinal product for snakebite treatment, so that their administration occurred prior to antivenom therapy at hospitals, it will be essential to evaluate different administration routes -- as topical, intravenous, and oral ones -- to verify their influences on outcomes.

In conclusion, rutin and RS exhibit different and direct activities towards BjV proteins. Pre-incubation of BjV with RS inhibited its coagulant and proteolytic activities, whereas pre-incubation with rutin did not show important changes *in vitro.* Otherwise, RS acted as an anticoagulant compound *in vitro*, possibly by its interaction with fibrinogen. *In vivo*, RS inhibited hemostatic disturbances partially, while local hemorrhage was completely blocked. Therefore, RS directly inhibits BjV SVMP *in vitro* and *in vivo*. Furthermore, rutin and RS showed important effectiveness on protecting mice from BjV toxicity, ensuring mice survival and improving hemostatic balance. Further studies are necessary to investigate the therapeutic potential rutin and RS in other envenomation models, and at different time intervals, as well as its use as a complementary agent to antivenom therapy.

## Data Availability

The raw data supporting the conclusion of this article will be made available by the authors, without undue reservation.
